# Psychological Health of Orphan Bonobos and Chimpanzees in African Sanctuaries

**DOI:** 10.1371/journal.pone.0017147

**Published:** 2011-06-07

**Authors:** Victoria Wobber, Brian Hare

**Affiliations:** 1 Department of Human Evolutionary Biology, Harvard University, Cambridge, Massachusetts, United States of America; 2 Department of Evolutionary Anthropology and Center for Cognitive Neuroscience, Duke University, Durham, North Carolina, United States of America; University of Utah, United States of America

## Abstract

**Background:**

Facilities across Africa care for apes orphaned by the trade for “bushmeat.” These facilities, called sanctuaries, provide housing for apes such as bonobos (*Pan paniscus*) and chimpanzees (*Pan troglodytes*) who have been illegally taken from the wild and sold as pets. Although these circumstances are undoubtedly stressful for the apes, most individuals arrive at the sanctuaries as infants and are subsequently provided with rich physical and social environments that can facilitate the expression of species-typical behaviors.

**Methods and Findings:**

We tested whether bonobo and chimpanzee orphans living in sanctuaries show any behavioral, physiological, or cognitive abnormalities relative to other individuals in captivity as a result of the early-life stress they experience. Orphans showed lower levels of aberrant behaviors, similar levels of average cortisol, and highly similar performances on a broad battery of cognitive tests in comparisons with individuals of the same species who were either living at a zoo or were reared by their mothers at the sanctuaries.

**Conclusion:**

Taken together, these results support the rehabilitation strategy used by sanctuaries in the Pan-African Sanctuary Alliance (PASA) and suggest that the orphans we examined did not show long-term signs of stress as a result of their capture. Our findings also show that sanctuary apes are as psychologically healthy as apes in other captive settings and thus represent a valuable resource for non-invasive research.

## Introduction

In the past 5 years, researchers have begun to study the behavior, cognition, endocrinology, morphology, health, and genetics of apes living in African sanctuaries [1]–[6]. The majority of the individuals at the sanctuaries are orphans of the “bushmeat trade,” having been confiscated as infants from poachers who kill and eat female chimpanzees and hope to sell their infants as pets [7]. The goal of the Pan-African Sanctuary Alliance (PASA) is to provide rich physical and social environments that allow individuals to recover from the stress they experience in being removed from their mother and from life in the wild [8], [9]. However, the degree to which sanctuary apes demonstrate species-typical behavior and psychology throughout their lives remains an empirical question [7], [10]. Given the potential for numerous research opportunities at the sanctuaries, it is important to assess the psychological health of these animals not only to inform management strategies but also to better characterize the sanctuary apes in relation to other captive populations participating in research. Previous work has investigated the effects of early-life stress on laboratory and zoo populations of chimpanzees (*Pan troglodytes*), assessing both social and environmental factors, but there has been little such research on bonobos (*Pan paniscus*), humans' other closest living relative. Accordingly, we review herein the findings for chimpanzees to provide a template for our study of psychological health in chimpanzees and bonobos living in sanctuaries.

### Factors affecting psychological health in captive apes

A number of previous studies have documented the effects of the rearing environment on the behavior of chimpanzees in captivity by using short-term studies of infants, retrospective studies of adults, or data on longitudinal development. The earliest of these studies were conducted on individuals who had been subjected to severe sensory and social isolation during nursery rearing that resulted in extreme levels of aberrant behaviors, including social and cognitive deficits that prevented these individuals from copulating, raising infants, or having a normal social life more generally [11]–[18]. These results demonstrated that chimpanzees who have been separated from their mother and peers and subjected to environmental deprivation suffer severe negative consequences.

More recent studies have investigated the impact of maternal separation and of rearing history in particular. In one, a large sample of chimpanzee infants (n = 46) who were rejected by their mothers within 3 months of birth were subsequently reared in a laboratory nursery, either interacting only with peers or being given additional interaction with a human surrogate mother. These two groups were then compared for attachment behaviors and cognitive abilities [19] (see also Maki and colleagues [20]). When tested before their first birthday, infants who had interacted only with peers possessed more disorganized attachment styles than infants who had been given human maternal care. However, both groups of infant chimpanzees performed similarly to human infants in the cognitive abilities measured, leading the authors to conclude, “…the current nursery chimpanzees did not experience severe deprivation and only chimpanzees raised in isolation suffer long-lasting and more severe deficits in cognition responses.” ([19], p. 181).

These findings were corroborated by a longitudinal study completed in 1996 that compared another group of laboratory infants taken from their mothers shortly after birth and raised in peer groups to a group of infants reared by their mothers [21], [22]. This study found that both populations expressed a full and normal range of social behaviors as adults. The author summarizes by saying, “…the unexpected conclusion to be drawn is that the separation followed by peer group rearing had little effect on the behavior observed. Body rocking and mouthing were the only behaviors that were influenced by [maternal] separation. No other unequivocal effect of deprivation [separation and absence of the mother] was found.” ([21], p. 73). Thus, this study suggested that even without care by human surrogates, chimpanzee infants reared with peers and given environmental stimulation can function normally as adults.

Finally, Bloomsmith et al [23] performed a review of the existing data to ascertain whether the age at which infant chimpanzees are separated from their mothers affects later psychological health. Laboratory orphans who lived with their mothers for at least 1 year before being rejected by their mothers showed little rocking behavior and four times as much maternal competence as adults than did individuals rejected by their mothers before the age of 1 year [23]. Moreover, laboratory infants orphaned after age 2 and then peer reared behaved similarly to mother-reared infants in subadulthood, leading the authors to conclude that 2 years of maternal rearing is enough to buffer chimpanzees against developing a variety of kinds of abnormal behavior [23].

Taken together, these studies indicate that laboratory chimpanzees are most likely to exhibit long-term behavioral abnormalities if they are separated from their mothers before age 2, housed in inadequate social environments without access to conspecific peers, or housed in a relatively sterile physical environment throughout their lives [23]. Importantly, these abnormal behaviors can also arise in adult chimpanzees who are singly housed, regardless of their rearing histories [24], [25]. However, even chimpanzees taken from their mothers and housed singly in a severely deprived environment throughout life can recover if introduced into a social group in late adulthood (i.e., in “retirement facilities”), with reductions in cortisol levels found as these individuals integrate into their new group (a reduction in cortisol correlates with reduced stress) [26]. This suggests that the factors essential to psychological health in any captive chimpanzees include: 1) the amount of time spent with the mother prior to separation (applicable only to orphans), 2) environmental stimulation, and 3) social housing. Below we discuss how African ape sanctuaries address these requirements of care for captive apes.

### Factors suspected to affect the psychological health of orphans in African ape sanctuaries

As discussed above, chimpanzee infants separated from their mothers at birth have a greater chance of showing behavioral aberrations than those who lived with their mothers for at least 1 year, although many infants separated at birth show few if any negative effects on their cognition when reared by their peers [19]. Apes who arrive at PASA sanctuaries are typically aged 2 to 3 years, according to dental and weight estimates made by sanctuary veterinarians based on published data [8]. This means that they were either removed from the wild early on in life and have lived in human care for several years, or were removed relatively recently and have only lived in human care for a short time. The former scenario presumes that the poachers who shot the orphan's mother or the individuals who bought the ape as a pet were able to successfully care for a neonatal chimpanzee. As nearly all of the individuals who arrive at the sanctuary are severely malnourished or dehydrated, it seems likely that apes taken from the wild as neonates would not survive the suboptimal care (if any) rendered by their captors and thus would never arrive at a sanctuary. Thus, this first possibility seems to be little more than hypothetical.

Alternatively, the infant may have lived with its mother for several years after birth and have lived with humans for only a short time prior to its arrival at the sanctuary. Such a scenario would increase the chances of an orphan's survival prior to its arrival at the sanctuary, and it seems like the only realistic path for those who survive to reach the sanctuary. It also suggests that sanctuary orphans lived in their mothers' care for a minimum of 1 year prior to being separated, with the majority likely in their mother's care for 2 or even 3 years. This indicates that orphans at the sanctuaries generally fit the laboratory models where individuals are separated from their mothers after several years of maternal care [23] rather than those where individuals are separated at birth [11], [19], [21], [22]. In addition, we should note that chimpanzee mothers in these laboratory studies frequently actively refused to care for their infants. Refusal to provide maternal care is often an indicator of poor status of the infant itself or poor psychological health of the mother (mothers with little experience with infants during their own development due to social deprivation tend to reject their own infants at high rates) [23]. In contrast, orphans at the sanctuaries were taken from, rather than rejected by, their mothers. Thus, we argue that sanctuary apes have likely received several years of quality maternal care prior to separation.

In terms of environmental stimulation, even mother-reared chimpanzees can develop numerous aberrant behaviors, such as coprophagy (eating feces) and regurgitation if they are not provided with adequately complex environmental resources in captivity [23]. However, after captive chimpanzees are supplied with material for wadging (a behavior in which they suck the juice out of solid foods) and bedding, these behaviors can disappear [27], [28]. The presentation of novel objects such as toys or uprooted trees can also lead to the reduction of these abnormal behaviors [29], [30]. Sanctuary apes have access to primary tropical forests every day (described in the Methods section below) that provide complexity and novelty unmatched by any laboratory or zoo facility. Thus, on this dimension the sanctuary environments may be better able than other captive facilities to meet the “top 10” requirements for the care of apes in captivity based on the daily choices that are available to them (Table 1) [31], [32].

**Table 1:** Top ten recommendations for the care and management of chimpanzees based on their behavior in the wild, and the means taken to satisfy them by typical U.S. laboratories and African sanctuaries.^a^

Recommendation	U.S. Laboratories	African Sanctuaries
1) Sites for elevated nesting and nesting material	Varied: not required by law	Elevated (4 m) sleeping hammocks with straw bedding
2) Space for sub-grouping and escape	Small non-forest enclosures	Large forest enclosures
3) Resources for foraging and processing rather than eating	Foraging enrichment program voluntary	Dozens of plant species available at all times for *ad libitum* foraging
4) Three-dimensional structures for travel and movement	Varies: zero to limited climbing structures of limited complexity	Primary tropical forest available all day with highest complexity
5) Equatorial photo periods (12 hr)	Seasonal/temperate climates	Equatorial photo periods
6) Mixed age and sex groups	Varies: mixed groups typical	All non-infant groups are mixed
7) Rivals and allies for dominance	Varies: multi-male groups available	All groups are multi-male
8) Community-level affliation	Yes, when socially housed	Always
9) Extended mother-offspring associations	Preferred, but high rejection rate among nursery-reared mothers	All raised by mothers in varying degrees before orphaned (0 to 5 years)
10) Mental stimulation characteristic of wild chimpanzees	Can be high in social domain but typically low in physical domain	High due to rich social and physical environment

a Adapted from Pruetz and McGrew (ref. 31) and Wrangham (ref. 32).

Finally, regarding social housing, chimpanzees who are singly housed can show considerable behavioral aberrations as a result [11], [18], [24], [25]. At the sanctuaries, chimpanzees and bonobos live in multi-male, multi-female groups that can form subgroups of varying size (a “fission-fusion” social system) due to the nature of their large forest enclosures (see Methods). When they are housed in smaller, less complex enclosures, in contrast, many captive groups of chimpanzees and bonobos do not have multiple adult males present or the opportunity to split into isolated subgroups. Thus, the social groups at African ape sanctuaries are likely a better approximation of social groupings in the wild than are those at other captive facilities.

In summary, previously published work on laboratory chimpanzees suggests that PASA orphans should not experience chronic trauma as a result of their early-life experience because they were mother reared for a significant period of time and are provided with highly complex social and physical environments at the sanctuary. In the present study we tested this hypothesis by assessing aberrant behaviors, levels of cortisol (a proxy for general physiological stress), and cognitive abilities in sanctuary orphans. In order to gauge their psychological health, we compared the sanctuary orphans to mother-reared apes living at the same sanctuaries and mother-reared individuals living in a zoo population. If the sanctuary management strategy allows orphaned individuals to develop relatively normally, they should be comparable to mother-reared individuals on these measures. Alternatively, if their early life experience leads to long-term psychological damage, then sanctuary orphans should show higher levels of behavioral aberrations and of average cortisol relative to mother-reared individuals while demonstrating less proficiency in cognitive tasks.

## Methods

For our first experiment, we examined several stereotyped behaviors observed in laboratory chimpanzees that are associated with social deprivation in infancy and low levels of environmental enrichment in adulthood (rocking, fecal smearing, and coprophagy) [25], [33]. We compared the rates of these behaviors in a group of sanctuary orphans and a group of zoo chimpanzees who were both anticipating social feeding (an arousing situation) [34]. In our second experiment, we investigated whether the early-life experiences of sanctuary orphans had any lasting effect on their average cortisol levels. Long-term physiological effects of early-life stressors have been observed in bonnet macaques (*Macaca radiata*) and common marmosets (*Callithrix jacchus*) that were nutritionally or socially deprived in infancy [35], [36]. We compared basal cortisol levels in orphan and mother-reared bonobos and chimpanzees, both living at the sanctuaries. Finally, in our third experiment, we investigated orphans' cognitive abilities relative to those of mother-reared individuals. While there is currently no evidence of cognitive deficit in chimpanzee infants removed from their mothers at birth, such separation can affect the cognitive abilities of human infants [37]. Thus, we compared the cognitive abilities of orphaned infants and mother-reared infants (both chimpanzee and bonobo) on a broad range of cognitive tasks designed to assess physical and social cognition (based on work by Herrmann and colleagues [38], [39], and by Tomasello and Carpenter [40]).

### Description of study sites

The two sanctuaries used for the current research are both members of PASA; Lola ya Bonobo is outside of Kinshasa in the Democratic Republic of the Congo, and the Tchimpounga Chimpanzee Rehabilitation Center is outside of Pointe Noire, Republic of the Congo. PASA has confiscated over 1,000 infants who were taken from their mothers as part of the illegal pet trade; these orphans currently live in 19 different orphanages or “sanctuaries” across sub-Saharan Africa [8], [9], [41], [42]. The goal of PASA is to rehabilitate these infants by providing them with the highest quality of lifetime care and the potential to be released back into the wild [9], [41], [43].

PASA has developed comprehensive management guidelines for the care of newly arrived infants to maximize the probability that initially distressed infants will grow into healthy adults [8]. When receiving an infant, the sanctuaries quarantine, examine, and treat the individual so that he/she can join a peer group as quickly as possible. After quarantine, infants are placed in a peer group of recent arrivals where they typically remain until they are strong enough to be integrated into a social group of mixed age and sex. After anywhere from 6 months to 2 years, infants are placed in these larger social groups of 10–30 individuals in which they live for the rest of their lives unless they are released back into the wild [9], [41], [43].

PASA sanctuaries meet certain standards of care in the facilities that they provide for the apes, and the two study sites meet all of the “top 10” suggestions for the care of captive chimpanzees [31], [32] (Table 1). All apes spend the night in dormitories that are similar to those found in any zoo, allowing for daily veterinary care [8], [9]. Also, much like a zoo, apes of all ages are released each morning to roam freely in outdoor enclosures. However, the sanctuary enclosures are larger and more diverse than those of a typical zoo or laboratory. They range in size from 5 to 40 hectares (a hectare is 10,000 square meters), and thus are 10–100 times the size of the largest existing zoo enclosures. Because of their size, the individuals living there can exhibit the species-typical behaviors of forming isolated subgroups (fission-fusion). The sanctuary enclosures also contain dozens of edible plants, trees for climbing and nesting, and small animals that are typically found in tropical forests, allowing a range of environmental complexity not feasible in other captive facilities.

In sum, once these apes arrive at the sanctuary, they live a life that more closely approximates that of a wild ape than what other individuals in captivity can experience. They are able to forage within large, multi-male–multi-female groups in substantial patches of food-producing forest.

## Results

### Experiment 1

In our first experiment, we assessed the levels of aberrant behavior in two groups of captive chimpanzees: one at the Wolfgang Köhler Primate Research Center at the Leipzig Zoo, Germany, and the second at the Tchimpounga Chimpanzee Rehabilitation Center. The zoo group consisted of 14 subjects (4 males and 10 females) and 3 dependent infants whom we did not include in the dataset because they were still being carried by their mothers. All of the adults were born and raised in a laboratory setting before arriving at the zoo as adults. All individuals in this group were mother reared. Throughout the year the zoo chimpanzees participate in a high level of environmental stimulation in the form of enrichment activities or cognitive problem-solving games. The Tchimpounga group consisted of 25 individuals (16 males and 9 females), all of whom were included in the dataset and were orphans of the bushmeat trade. This population participates in enrichment games for just a few months of the year but has access to a tropical forest enclosure. The mean ages of the zoo and sanctuary groups did not differ (independent samples t-test, p>0.3) with means of 18.8 years (range: 4–31) in the zoo group and 18.4 years (range: 11–39) in the Tchimpounga group. Data was collected before the groups were released into their sleeping rooms, where a large quantity of sharable food was placed (the evening meal). The zoo chimpanzees were observed during the winter in a large indoor enclosure (0.43 hectares), while the Tchimpounga chimpanzees were observed while they were in a 25-hectare forested enclosure. Subjects were not food deprived in any way for the purpose of this test.

At both sites, nine food-sharing trials were performed over the course of 1 month (January 2007). Behaviors of all visible individuals were recorded every 10 minutes using scan sampling ([44], p. 90). In the zoo group, the start and finish time of this nightly pre-feeding session were consistent so that individuals were always observed for 50 minutes prior to feeding. For the Tchimpounga group, the end of this pre-feeding time varied slightly, and so the observations lasted either 40, 50, or 60 minutes depending on when the subjects were released into their sleeping enclosure. As a result of the variability, there were 46 total scans at Tchimpounga versus 45 in the zoo.

At each of the 10-minute intervals, the observer noted the behavior of each subject, systematically scanning left to right to determine whether he/she was exhibiting one of the following behaviors, with only one behavior scored on any given scan (as defined by Walsh and colleagues [33] and Goodall [45]):

**Aberrant behaviors.** *Rocking*: rhythmically moving forward/backward or side to side; *Coprophagy*: ingestion of feces; and *Fecal smearing*: spreading of feces on a surface with the hands or mouth.

**Species-typical behaviors.** *Social grooming*: use of both hands to part the hair of a conspecific while picking at that individual's exposed skin with lips, thumb, or index finger, and *Eating:* zoo chimpanzees could feed on items in puzzle boxes inside their enclosure, while sanctuary chimpanzees could feed on the plant matter in their enclosure.

The three aberrant behaviors were selected because they are the most frequent aberrations that occur in laboratory populations where individuals have grown up in suboptimal conditions or currently live in such conditions [33]. The species-typical behaviors were recorded as basic behavioral markers of activities that occur during a chimpanzee's normal daily routine of socializing and foraging [45]. If the subject was visible but not engaged in any of these behaviors, he/she was scored only as “present.”

The number of individual data points differed between the zoo and Tchimpounga groups because all of the zoo individuals were always visible, resulting in 45 observations for each of these individuals. In contrast, at Tchimpounga, individuals may have been hidden in the forest, and thus the number of observations varied between individuals (range: 6–46). To take this into account, percentages of the total number of samples where an individual was visible in which the individual engaged in a given behavior were calculated and compared across groups. Thus, if an individual was present at 38 scans and engaged in coprophagy during 1 of these 38, 2.63% was noted as her/his percentage of scans engaging in coprophagy. The percentages for each subject of the behaviors observed did not necessarily add up to 100%, as subjects could have been engaging in another activity (walking, resting) that was not scored as part of this experiment. Nonparametric statistics were used, and all p-values reported are 2-tailed.

**Figure 1:**
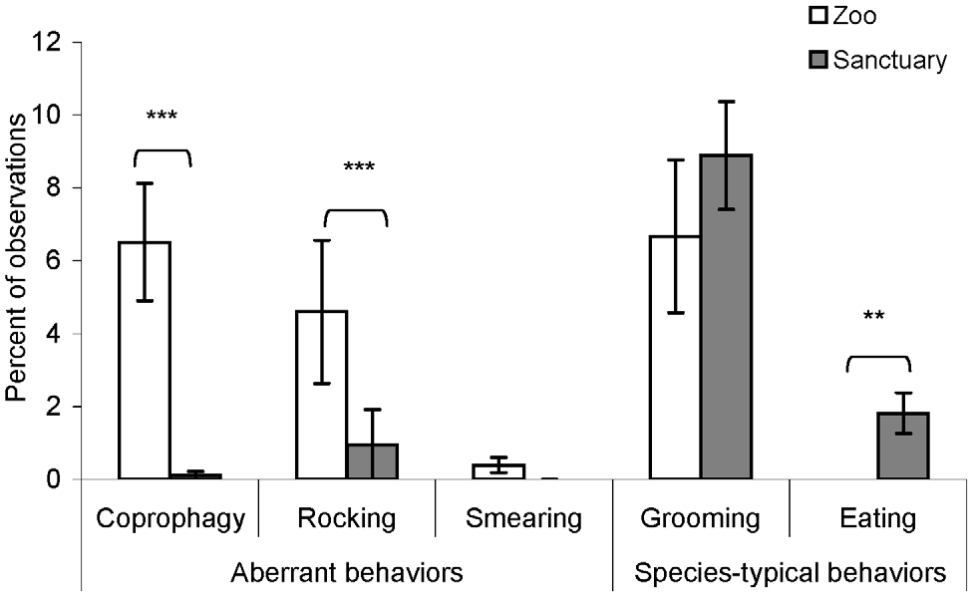
Average percentage of scans spent by zoo and sanctuary individuals in each behavior, experiment 1. The error bars represent the standard error of the mean. Sanctuary individuals exhibited significantly lower levels of two aberrant behaviors (coprophagy and rocking) while showing similar or even greater levels of species-typical behaviors in comparison to the zoo population. Significant differences between groups are represented with ** p≤0.01, *** p≤0.001.

There were significant differences between the zoo and sanctuary groups in percentage of scans where they engaged in coprophagy (Mann-Whitney U, Z = −5.100, asymptotic significance, p<0.001), rocking (Z = −3.196, asymptotic significance, p = 0.001), and eating (Z = −2.499, asymptotic significance, p = 0.012) (Figure 1). Zoo individuals exhibited more coprophagy and rocking, while sanctuary individuals exhibited more eating. Fecal smearing occurred so rarely that statistics could not be performed for it; the behavior was observed three times at the zoo and never at the sanctuary. There were no significant differences between groups in rates of grooming (Figure 1).

There were several significant differences between groups in the percentage of individuals exhibiting a particular behavior at least once (Table 2). For example, a significantly greater proportion of the zoo individuals exhibited coprophagy at least once: 12 of 14 in the zoo versus 1 of 25 in the sanctuary (Pearson chi-square = 26.966, p<0.001). Seven of 14 individuals at the zoo displayed rocking at least once, as opposed to only 1 of 25 at the sanctuary (a different individual from the one exhibiting coprophagy) (chi-square = 11.647, p = 0.001). Three of 14 individuals exhibited fecal smearing in the zoo population, whereas no individual did so at the sanctuary (chi-square = 5.804, p = 0.020). The number of individuals exhibiting any grooming was more comparable between the groups, with 9 of 14 zoo individuals grooming another group member while 20 of the 25 sanctuary individuals did so (chi-square = 1.162, p = not significant). Finally, 9 of 25 individuals at the sanctuary fed during the observation period, whereas no individuals did so in the zoo (chi-square = 6.552, p = 0.010). Therefore, we found little evidence for the most commonly observed aberrant behaviors in the Tchimpounga sanctuary chimpanzees, with this population showing even fewer of these behaviors than zoo chimpanzees that participate in an active enrichment program and live in one of the largest ape enclosures in Europe.

**Table 2:** Percentage of individuals in each population (sanctuary and zoo) exhibiting a given behavior at least once in the pre-feeding context, experiment 1.

	Aberrant behaviors			Species-typical behaviors	
	Coprophagy	Rocking	Smearing	Grooming	Eating
Zoo (n = 14)	85.7***	50.0***	21.4*	64.3	0.0
Sanctuary (n = 25)	4.0	4.0	0.0	80.0	36.0**

Significant differences between groups are represented with

*p≤0.05,

**p≤0.01,

***p≤0.001.

### Experiment 2

For our second experiment, we collected saliva samples from six mother-reared individuals (four chimpanzees and two bonobos) at the two sanctuaries described above, ranging in age from 3 to 12 years (average: 5.3 years) and consisting of five males and one female. We compared their average cortisol levels to those of six species-, sex-, and age-matched (where possible) orphans at the sanctuaries (four chimpanzees and two bonobos with an average age of 5.7 years, five males and one female). Because all subjects lived at the sanctuaries they were fed the same diet, although some of the younger mother-reared individuals were still receiving some breast milk. Thus as to the concern that cortisol levels might be affected by differences in nutritional status, all individuals were well fed and not injured or visibly ill when the samples were taken.

Cortisol levels were assessed using radioimmunoassay of saliva samples; the sample collection and laboratory analysis are described elsewhere [34], [46]. At least two samples were collected from each individual, with each sample collected on a separate day (range: 2 to 8 per individual, average of 5.7). Samples were all collected during daytime hours (range: 8:48 AM to 4:49 PM), and collection within a given individual was balanced in terms of collecting an equal number of morning and afternoon samples when possible. We also controlled for circadian rhythms to some extent by avoiding sampling during the early morning, when cortisol peaks (chimpanzees and bonobos at the sanctuaries awake when the sun rises, at approximately 6:00 AM). Samples were collected in the summer of 2008 and analyzed in the fall of that year. Nonparametric statistics were used, and all p-values reported below are 2-tailed.

**Figure 2:**
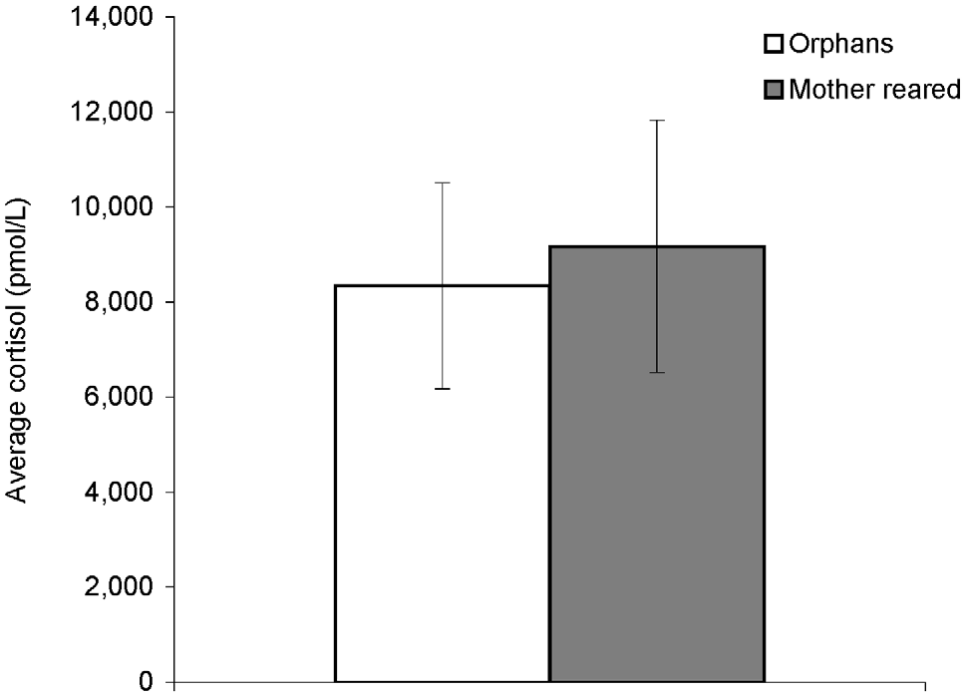
Average cortisol in mother-reared and orphan individuals at the sanctuaries, experiment 2. Individual averages were based on 2 to 8 samples per individual, and statistical analyses were performed with these individual averages. Cortisol levels did not differ significantly between the two groups (Mann-Whitney U). As these groups were sampled at a similar time of day and fed similar diets, this suggests that orphans do not exhibit markers of significant psychological stress relative to mother-reared individuals.

Comparisons of individual averages between mother-reared individuals and orphans revealed no differences in cortisol levels (average values were 9,170 pmol/L for mother-reared individuals and 8,340 pmol/L for orphans, Mann-Whitney U, Z = 0.160, p = 0.87) (Figure 2). The amount of individual variation also did not differ between groups, as measured by the standard error of an individual's samples around that individual's average level (average standard errors were 2,350 pmol/L for mother-reared individuals and 2,120 pmol/L for orphans, Mann-Whitney U, Z = 0.801, p = 0.42). Sampling of the two groups was highly similar in that the number of samples per individual did not differ significantly between the two groups (average for mother reared, 5.0; for orphans, 6.3), nor did the average time of collection (average for mother reared was 12:34 PM; for orphans, 11:38 AM). Therefore, our comparison provides no evidence that sanctuary orphans' cortisol levels are different than those of individuals who were born at the sanctuary and reared by their mothers.

### Experiment 3

For this experiment, we again compared mother-reared and orphan chimpanzees and bonobos, including both sanctuary and zoo individuals in our sample. We tested 14 mother-reared infants: 3 were chimpanzees from Tchimpounga; 6 were bonobos from Lola ya Bonobo; and 5 were from the Leipzig Zoo (4 chimpanzees and 1 bonobo). We also tested 7 orphan chimpanzees at Tchimpounga and 7 orphan bonobos at Lola ya Bonobo who matched the age and sex of the mother-reared individuals as best as possible. Among the 14 mother-reared individuals, there were 9 males and 5 females, with a mean age of 2.8 years. The orphan group consisted of 7 males and 7 females, with a mean age of 3.1 years (no significant difference in age, independent samples t-test, p>0.5). Here we used parametric statistical analyses, and all p-values reported are 2-tailed. Because 2 of the 14 mother-reared individuals did not complete the majority of the physical cognition tasks, 2 orphans from the 14 tested were removed from the analyses to make the sample sizes as comparable as possible.

As in experiment 1, subjects were not food deprived for testing, and water was always available. Subjects were tested in their dormitories in rooms that were familiar to them. In all cases, an individual's mother or human caretaker was in the same room or a neighboring room, and all testing was voluntary. If subjects indicated they were uncomfortable by refusing to eat or by sitting near the exit to the testing room, the session was stopped. We should note that sanctuary apes participate in these short test sessions so rarely that playing our games remains a special treat (sanctuary apes were tested a mean of 7 days per year in 2008 when this data was collected). Individuals were tested between June 2008 and January 2009.

We tested subjects on a subset of the Primate Cognition Test Battery (PCTB) [38], [39] that had been adapted for a developmental sample by including four additional tasks designed for infants [40] (see also forthcoming article by the first author and colleagues). In the present battery, nine tasks involved social cognition and five concerned physical cognition. Four of the social cognition tasks and all five physical cognition tasks were taken from the PCTB. The four social cognition tasks from the PCTB were social learning (one item only), attentional state, gaze following, and intentions, while the five tasks involving physical cognition from that battery were object permanence, transposition, relative numbers, tool use, and tool properties. The procedures for these tasks were performed exactly as described in supplemental material within Herrmann and associates 2007 [38]. Three of the remaining social cognition tasks were taken from a published battery of tasks investigating sociocognitive development in human and chimpanzee infants [40]; these were gaze following around barriers, social obstacle, and intention emulation. Again, the methods were carried out as described in this previous work [40]. One additional social cognition task used in our previous research was added, measuring social inhibitory control [3]. The methods of the ninth social cognition task, reputation, are described below.

The general setup of all tasks was the same regardless of a subject's rearing history or testing environment (zoo or sanctuary). Individuals interacted with an experimenter who was separated from them by the mesh bars or Plexiglas plates of the dormitory walls. The experimenter typically sat behind a table and presented subjects with situations where they could obtain a food reward for correct performance. All tests were videotaped. Performance on tasks where scoring could be ambiguous (e.g., where subjects were not simply making a choice between two options) was coded for reliability by staff who were blind to the hypotheses of the study. A random 20% of the videotapes for these tasks were selected; reliability was high (Cohen's kappa of 0.67 or greater).

For the reputation task, three experimenters stood outside the mesh or Plexiglas wall of the testing room; each of the three had a designated role as “nice,” “mean,” or “neutral.” To begin each trial, the neutral experimenter called the subject's name. The experimenters then began a demonstration in which the nice experimenter attempted to give a piece of food to the neutral experimenter, but the mean experimenter took it away and ate it (or simulated eating it). This demonstration was repeated 10 times. The nice and mean experimenters then left the testing area, and each took 10 peanuts out of a bowl. They returned to the testing area and stood 2 meters apart at the mesh/Plexiglas wall. The neutral experimenter presented the subject with a peanut at the mesh in the middle of these two experimenters so that the subject would be equidistant from the two experimenters before its choice. The experimenters then presented their hands face up to the subject so that the subject could see they were holding peanuts and could beg from one or both of them. The experimenters held their hands out for 20 seconds but did not reward the subject if it approached. The entire procedure (10 demonstrations and a choice of whom to beg from) was then repeated three times, for a total of four trials. The dependent measure in this task was the proportion of trials where subjects made a choice in which the subject approached the nice experimenter first.

To complete the task battery, subjects participated in multiple testing sessions, which were presented once a day and lasted approximately 30 minutes. Individuals completed the battery in 7 to 10 test sessions (depending on their level of motivation).

**Figure 3:**
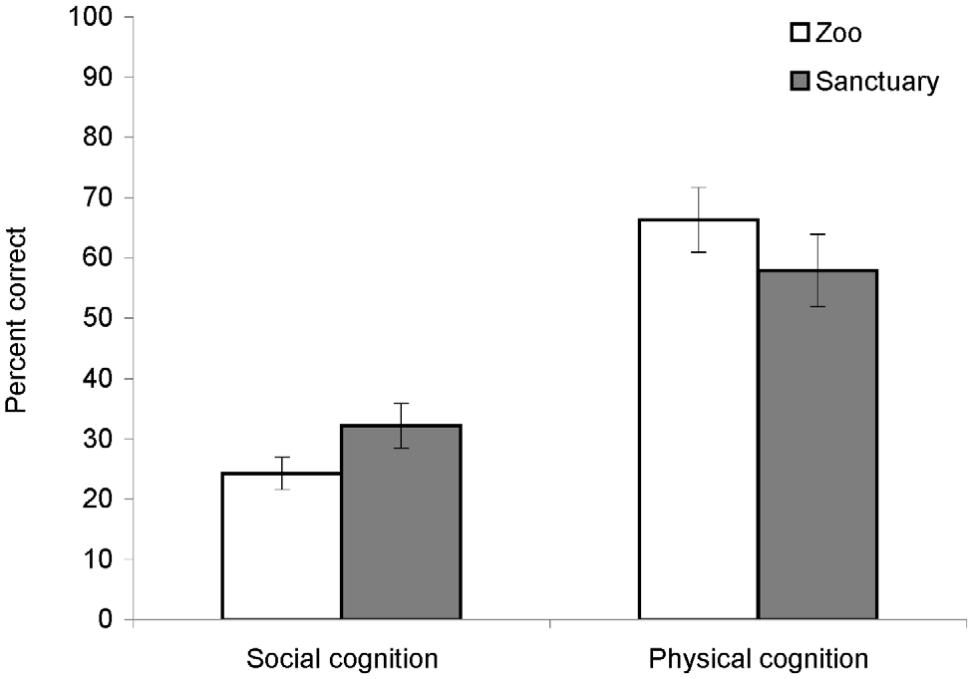
Average percentage of correct choices in cognitive tasks in mother-reared individuals, by environment, experiment 3. Bars denote standard error. Mother-reared individuals in the zoo and in the sanctuary performed no differently in social cognition or physical cognition tasks. The n = 9 sanctuary individuals and 5 zoo individuals for social cognition, and n = 7 sanctuary individuals and 5 zoo individuals for physical cognition, as not all individuals completed both domains of tests.

Performance on these tasks was recorded as the average of the nine social cognition tasks and the average of the five physical cognition tasks. We first performed independent samples t-tests for each of these domains with the mother-reared individuals to determine whether living in a zoo or sanctuary environment affected performance. We found no effect of this variable on social cognition (n = 9 sanctuary, n = 5 zoo, p>0.1) or physical cognition (n = 7 sanctuary, n = 5 zoo, p>0.3) (Figure 3), suggesting that mother-reared infants performed just as well when raised in a sanctuary or in a zoo. We thus combined these mother-reared individuals to compare them with the orphans at the sanctuaries. Independent samples t-tests demonstrated no effect of mother rearing on performance in social cognition (n = 14 mother reared, n = 14 orphan, p>0.7), but a significant effect of mother rearing was found for physical cognition (n = 12 mother reared, n = 12 orphan, t(22) = 2.75, p = 0.01), with mother-reared individuals outperforming orphans. However, analyses performed for each individual cognitive task (independent samples t-tests for tasks where performance was measured as the percentage correct and chi-squared tests where a success/failure variable was used) revealed that there was a significant difference between mother-reared and orphan infants in only one task, tool properties (t(22) = 2.04, p = 0.05), where mother-reared infants performed better (Table 3). On one other task, tool use, there was a tendency toward more skilled performance by mother-reared infants, but the difference did not quite reach significance (χ^2^ (1, n = 24) = 3.70, p = 0.06). Thus, while tool use in particular may be sensitive to maternal contact (see [47], [48], [49] for individual differences in the acquisition of tool competency), our findings indicate that orphans perform similarly to mother-reared individuals on the vast majority (13 out of 14) of cognitive tasks spanning both physical and social cognition.

**Table 3:** Average percentage of correct choices in each of the cognitive tasks according to rearing history, experiment 3.

	Orphan		Mother-reared		
Task	Mean	n	Mean	n	Significantly different?
Intention emulation	0.21	14	0.00	8	No
Social obstacle	0.15	13	0.27	10	No
Gaze-following around barriers	0.21	14	0.23	13	No
Social inhibition	0.52	13	0.43	13	No
Gaze-following	0.22	14	0.25	14	No
Attentional state	0.20	13	0.10	14	No
Intentions	0.64	13	0.57	12	No
Social learning	0.07	14	0.00	8	No
Reputation	0.56	14	0.57	14	No
Object permanence	0.58	12	0.68	12	No
Transposition	0.44	12	0.57	12	No
Relative numbers	0.72	12	0.72	12	No
Tool properties	0.53	12	0.63	12	Yes (p = 0.05)
Tool use	0.08	12	0.44	9	No

The first 9 items are social cognition tasks and the latter 5 are physical cognition tasks.

## Discussion

We found little evidence that orphan apes in PASA sanctuaries exhibit any long-term consequences of their capture and removal from the wild early in life. The findings of all three of our experiments strongly support the hypothesis that the management strategies used by PASA sanctuaries allow orphan apes to develop species-typical behavior, physiology, and cognitive skills. In our first experiment we found that sanctuary chimpanzees had very low rates of coprophagy, fecal smearing, and rocking, three aberrant behaviors that are often expressed at high frequency in environmentally or socially deprived populations of captive chimpanzees [25], [33]. Indeed, the sanctuary chimpanzees exhibited lower rates of coprophagy and rocking than chimpanzees in a modern zoo facility with an active enrichment program, rich social life, and spacious enclosures, and no fecal smearing at all was observed among the sanctuary group. In our second comparison we found that mother-reared chimpanzees and bonobos had cortisol levels similar to those of orphans matched for species, age, and sex. Because the orphan and mother-reared individuals received comparable diets and were all healthy at the time of sampling, these results suggest that orphans at the sanctuaries are under no greater psychological stress than mother-reared individuals [50]. Finally, in our third experiment, sanctuary orphans showed social cognition abilities comparable to those for same-aged mother-reared infants and were less skillful in only one of five physical cognition tasks. Moreover, mother-reared infants living in the sanctuaries and in the zoo population performed similarly. Therefore, the results of these three experiments demonstrate that apes being rehabilitated at these two PASA sanctuaries are best characterized as psychologically healthy relative to other captive apes, even as infants.

While all three of our experiments found few differences between the sanctuary and zoo populations, it is possible that future work using more sensitive measures will identify a greater number of differences. For example, a comparison of laboratory chimpanzees found that adult nursery-reared females experienced more wounding in complex social settings but not in smaller social groups [51]. It may be that specific subpopulations of sanctuary apes do not cope as well socially as mother-reared individuals in certain contexts. In addition, it is widely known that early life stress can have effects on growth, endocrine function, and the immune system. Therefore, longitudinal research will be ideal, when feasible, to more fully test for the potential effects of sanctuary orphans' early-life experiences across an even wider set of phenotypic variables. Studies of cortisol reactivity can assess whether orphans are more vulnerable to environmental stressors than mother-reared individuals. There is currently little data with any population of ape (captive or wild) to address this question. In traits where previous non-human primate welfare research has found the greatest detrimental effects of a suboptimal living environment, we found little difference between individuals living at African ape sanctuaries and those living in a highly enriched zoo facility.

We should also note that we did not examine aberrant behaviors in bonobos. While informal observation and interviews of senior sanctuary staff indicate the same low frequency of aberrant behaviors in the sanctuary bonobos as seen in the sanctuary chimpanzees, it will be important to develop an ethogram specific to bonobos and conduct a direct comparison of sanctuary and zoo bonobos as well. However, we suspect that PASA sanctuaries caring for chimpanzees, bonobos, and gorillas are able to successfully rehabilitate orphans of the bushmeat trade whenever those infants survive the initial quarantine period.

Another potential limitation of our study is that we have quantitatively compared sanctuary apes only to other captive apes rather than to wild ape populations. Comparisons of wild and captive populations are rare, though possible in the case of endocrine measures and observational studies. For the study of psychology in particular, the paucity of such comparisons is largely due to the methodological differences between research with apes in captivity and in the wild. Experimental manipulations are essential to understand the cognitive mechanisms underlying complex behaviors. Yet experiments have rarely been viewed as a feasible or ethical way to study wild apes (many experimental techniques require food provisioning, etc.). Playback experiments and research on naturally occurring tool use represent exceptions, but in neither case have quantitative comparisons between captive and wild apes ever been reported [47], [48], [52], [53]. Therefore, only qualitative comparisons are currently possible between captive and wild apes in the area of psychology.

Such qualitative comparisons suggest that captive and wild populations of apes are largely psychologically similar. First, the low levels of aberrant behavior exhibited by the sanctuary chimpanzee population in our first experiment is more similar to the infrequent observations of these behaviors in wild chimpanzees than to the high levels observed in certain laboratory populations. In addition, a recent qualitative observational study documenting the diversity of tool-using behavior in zoo, sanctuary and wild ape populations showed a high degree of similarity between these populations [53]. Further, captive apes often show skills not observed in wild apes (e.g. instrumental cooperation in bonobos and female chimpanzees) [54], [55]. Finally, the strongest evidence that sanctuary bonobos and chimpanzees are psychologically similar to wild apes is the fact that rehabilitated orphans of both species have been successfully released back into the wild – including individuals from the sanctuaries involved in the current research [9], [41], [43]. Initial comparisons of activity budgets in released orphans and wild populations show few differences between the two [56]. Further direct quantitative comparisons between wild and captive populations, where possible, will help to build on the conclusions drawn from these generally qualitative comparisons.

Although we found strong evidence that sanctuary orphans are psychologically healthy, in no way should our results be construed to suggest that the capture or removal of infant chimpanzees and bonobos from their mothers is justified in any context. There is substantial evidence to suggest that chimpanzees removed from their mothers in early infancy do suffer intensely in the short term and can experience behavioral problems throughout life [23], [24], [57], [58]. In stark contrast, sanctuary orphans are raised by their mothers in early life and offered the highest level of captive care upon arriving at the sanctuary. Thus, we fully agree that in all cases where orphaning can be prevented, apes should be reared by their mothers [51], [58].

Overall, then, while sanctuary apes are often found in substandard conditions before their arrival at the sanctuary, there is currently little evidence of lasting behavioral or psychological damage as a result. Our findings corroborate previous indications that adult bonobo and chimpanzee orphans perform as well as or better than zoo apes in several cognitive tasks [54], [55], [59], [60]. These findings also provide an explanation for the high survival rate of bonobos and chimpanzees released back into the wild after having been rehabilitated at PASA sanctuaries [9], [41], [43]. Thus, research with sanctuary apes represents a near-unique opportunity to experimentally study cognition in captive apes living in an evolutionarily relevant environment. Further, research at African ape sanctuaries directly supports conservation efforts in ape-range countries. Through collaboration with the active education programs at the sanctuaries, research at these facilities can help to ultimately reduce the number of ape infants being taken from the wild.

## References

[1] Horner V, Whiten A (2005). Causal knowledge and imitation/emulation switching in chimpanzees (*Pan troglodytes*) and children (*Homo sapiens*).. Anim Cogn.

[2] Pika S, Zuberbühler Z (2008). Social games between bonobos and humans: evidence for shared intentionality?. Am J Primatol.

[3] Wobber V, Wrangham R, Hare B (2010). Bonobos exhibit delayed development of social behavior and cognition relative to chimpanzees.. Curr Biol.

[4] McIntyre MH, Herrmann E, Wobber V, Halbwax M, Mohamba C (2009). Bonobos have a more human-like second-to-fourth finger length ratio (2D∶4D) than chimpanzees: a hypothesized indication of lower prenatal androgens.. J Hum Evol.

[5] Mugisha L, Pauli G, Opuda-Asibo J, Joseph OO, Leendertz FH (2010). Evaluation of poliovirus antibody titers in orally vaccinated semi-captive chimpanzees in Uganda.. J Med Primatol.

[6] Krief S, Escalante AA, Pacheco MA, Mugisha L, Andre C (2010). On the diversity of malaria parasites in African apes and the origin of *Plasmodium falciparum* from Bonobos.. PLoS Pathog.

[7] Beck B, Lonsdorf E, Ross S, Matsuzawa T (2010). Chimpanzee orphans: sanctuaries, reintroduction, and cognition.. The mind of the chimpanzee: Ecological and experimental perspectives.

[8] Cox D, Rosen N, Montgomery C, Seal U (2000). Chimpanzee Sanctuary Guidelines and Management Workshop: Report.

[9] Farmer KH (2002). Pan-African Sanctuary Alliance: status and range of activities for great ape conservation.. Am J Primatol.

[10] Wrangham R, Lonsdorf E, Ross T, Matsuzawa T (2010). Afterword: meanings of chimpanzee mind.. The mind of the chimpanzee: Ecological and experimental perspectives.

[11] Davenport RK, Menzel EW (1963). Sterotyped behavior of the infant chimpanzee.. Arch Gen Psychiatry.

[12] Davenport RK, Rogers CM (1968). Intellectual performance of differentially reared chimpanzees: I. Delayed response.. Am J Ment Defic.

[13] Davenport R, Menzel E, Bourne G (1970). Differential rearing of the chimpanzee: a project survey.. The chimpanzee. 3^rd^ Edition.

[14] Davenport RK, Rogers CM, Menzel EW (1969). Intellectual performance of differentially reared chimpanzees: II. Discrimination-learning set.. Am J Ment Defic.

[15] Davenport RK, Rogers CM, Rumbaugh DM (1973). Long-term cognitive deficits in chimpanzees associated with early impoverished rearing.. Dev Psychol.

[16] Menzel EW, Davenport RK, Rogers CM (1970). The development of tool using in wild-born and restriction-reared chimpanzees.. Folia Primatol.

[17] Turner CH, Davenport RK, Rogers CM (1969). The effect of early deprivation on the social behavior of adolescent chimpanzees.. Am J Psychiatry.

[18] Rogers CM, Davenport RK (1971). Intellectual performance of differentially reared chimpanzees. 3. Oddity.. Am J Ment Defic.

[19] van Ijzendoorn MH, Bard KA, Bakermans-Kranenburg MJ, Ivan K (2009). Enhancement of attachment and cognitive development of young nursery-reared chimpanzees in responsive versus standard care.. Dev Psychobiol.

[20] Maki S, Fritz J, England N (1993). An assessment of early differential rearing conditions on later behavioral development in captive chimpanzees.. Infant Behav Dev.

[21] Spijkerman R (1996). Effects of peer-only rearing in young chimpanzees: a comparison of social behavior development in peer and family groups.

[22] Spijkerman RP, van Hooff JARAM, Dienske H, Jens W (1997). Differences in subadult behaviors of chimpanzees living in peer groups and in a family group.. Int J Primatol.

[23] Bloomsmith M, Baker K, Ross S, Lambeth S, Sackett G, Ruppenthal G (2005). Early rearing conditions and captive chimpanzee behavior: some surprising findings.. Nursery rearing of nonhuman primates in the 21st century.

[24] Baker KC (1996). Chimpanzees in single cages and small social groups: effects of housing on behavior.. Contemp Top Lab Anim Sci.

[25] Nash LT, Fritz J, Alford PA, Brent L (1999). Variables influencing the origins of diverse abnormal behaviors in a large sample of captive chimpanzees (*Pan troglodytes*).. Am J Primatol.

[26] Reimers M, Schwarzenberger F, Preuschoft S (2007). Rehabilitation of research chimpanzees: stress and coping after long-term isolation.. Horm Behav.

[27] Baker KC, Easley SP (1996). An analysis of regurgitation and reingestion in captive chimpanzees.. Appl Anim Behav Sci.

[28] Fritz J, Maki S, Nash LT, Martin T, Matevia M (1992). The relationship between forage material and levels of coprophagy in captive chimpanzees (*Pan troglodytes*).. Zoo Biol.

[29] Maki S, Bloomsmith MA (1989). Uprooted trees facilitate the psychological well-being of captive chimpanzees.. Zoo Biol.

[30] Paquette D, Prescott J (1988). Use of novel objects to enhance environments of captive chimpanzees.. Zoo Biol.

[31] Pruetz J, McGrew W, Brent L (2001). What does a chimpanzee need? Using natural behavior to guide the care and management of captive populations.. Care and management of captive chimpanzees.

[32] Wrangham R, Erwin J (1992). Living naturally: aspects of wild environments relevant to captive chimpanzee management.. Chimpanzee conservation and public health: Environments for the future.

[33] Walsh S, Bramblett CA, Alford PL (1982). A vocabulary of abnormal behaviors in restrictively reared chimpanzees.. Am J Primatol.

[34] Wobber V, Hare B, Maboto J, Lipson S, Wrangham R (2010). Differential changes in steroid hormones before competition in bonobos and chimpanzees.. Proc Natl Acad Sci U S A.

[35] Stevens HE, Leckman JF, Coplan JD, Suomi SJ (2009). Risk and resilience: early manipulation of macaque social experience and persistent behavioral and neurophysiological outcomes.. J Am Acad Child Adolesc Psychiatry.

[36] Law AJ, Pei Q, Walker M, Gordon-Andrews H, Weickert CS (2009). Early parental deprivation in the marmoset monkey produces long-term changes in hippocampal expression of genes involved in synaptic plasticity and implicated in mood disorder.. Neuropsychopharmacology.

[37] Beckett C, Maughan B, Rutter M, Castle J, Colvert E (2006). Do the effects of early severe deprivation on cognition persist into early adolescence? Findings from the English and Romanian adoptees study.. Child Dev.

[38] Herrmann E, Call J, Hernandez-Lloreda MV, Hare B, Tomasello M (2007). Humans have evolved specialized skills of social cognition: the cultural intelligence hypothesis.. Science.

[39] Herrmann E, Hernandez-Lloreda MV, Call J, Hare B, Tomasello M (2010). The structure of individual differences in the cognitive abilities of children and chimpanzees.. Psychol Sci.

[40] Tomasello M, Carpenter M (2005). The emergence of social cognition in three young chimpanzees.. Monogr Soc Res Child Dev.

[41] Andre C, Kamate C, Mbonzo P, Morel D, Hare B, Takesi I, Thompson J (2008). The conservation value of Lola ya Bonobo Sanctuary.. Bonobos revisited: Ecology, behavior, genetics, and conservation.

[42] Wrangham R (2008). The International Primatological Society as a coalition: primatologists and the future of primates.. Int J Primatol.

[43] Tutin CEG, Ancrenaz M, Paredes J, Vacher-Vallas M, Vidal C (2001). Conservation biology framework for the release of wild-born orphaned chimpanzees into the Conkouati Reserve, Congo.. Conserv Biol.

[44] Martin P, Bateson P (1986). Measuring behavior.

[45] Goodall J (1986). The chimpanzees of Gombe: Patterns of behavior.

[46] Lipson SF, Ellison PT (1989). Development of protocols for the application of salivary steroid analyses to field conditions.. Am J Hum Biol.

[47] Matsuzawa T, Biro D, Humle T, Inoue-Nakamura N, Tonooka R, Matsuzawa T (2001). Emergence of culture in wild chimpanzees: education by master–apprenticeship.. Primate origins of human cognition and behavior.

[48] Biro D, Sousa C, Matsuzawa T, Matsuzawa T, Tomonaga M, Tanaka M (2006). Ontogeny and cultural propagation of tool use by wild chimpanzees at Bossou, Guinea: Case studies in nut cracking and leaf folding.. Cognitive development in chimpanzees.

[49] Lonsdorf EV (2006). What is the role of mothers in the acquisition of termite-fishing behaviors in wild chimpanzees (*Pan troglodytes schweinfurthii*)?. Anim Cogn.

[50] Sapolsky R (1994). Why zebras don't get ulcers.

[51] Baker KC, Seres M, Aureli F, de Waal FBM (2000). Evaluating social enrichment of chimpanzees: injury risks under three housing conditions.. Am J Primatol.

[52] Wilson M, Hauser M, Wrangham R (2001). Does participation in intergroup conflict depend on numerical assessment, range location, or rank for wild chimpanzees?. Anim Behav.

[53] Gruber T, Clay Z, Zuberbuhler K (2010). A comparison of bonobo and chimpanzee tool use: evidence for a female bias in the *Pan* lineage.. Anim Behav.

[54] Melis AP, Hare B, Tomasello M (2006). Chimpanzees recruit the best collaborators.. Science.

[55] Hare B, Melis AP, Woods V, Hastings S, Wrangham R (2007). Tolerance allows bonobos to outperform chimpanzees on a cooperative task.. Curr Biol.

[56] Farmer K, Buchanan-Smith H, Jamart A (2006). Behavioural Adapatation of *Pan troglodytes*.. Int J Primatol.

[57] Kalcher E, Franz C, Crailsheim K, Preuschoft S (2008). Differential onset of infantile deprivation produces distinctive long-term effects in adult ex-laboratory chimpanzees (*Pan troglodytes*).. Dev Psychobiol.

[58] Matsuzawa T, Tomonaga M, Tanaka M (2006). Cognitive development in chimpanzees.

[59] Vlamings PHJM, Hare B, Call J (2010). Reaching around barriers: the performance of the great apes and 3–5-year-old children.. Anim Cogn.

[60] Hanus D, Call J (2008). Chimpanzees infer the location of a reward on the basis of the effect of its weight.. Curr Biol.

